# The Right Planum Temporale Is Involved in Stimulus-Driven, Auditory Attention – Evidence from Transcranial Magnetic Stimulation

**DOI:** 10.1371/journal.pone.0057316

**Published:** 2013-02-20

**Authors:** Marco Hirnstein, René Westerhausen, Kenneth Hugdahl

**Affiliations:** 1 Department of Biological and Medical Psychology, University of Bergen, Bergen, Norway; 2 Division of Psychiatry, Haukeland University Hospital, Bergen, Norway; 3 Department of Radiology, Haukeland University Hospital, Bergen, Norway; University of Salamanca- Institute for Neuroscience of Castille and Leon and Medical School, Spain

## Abstract

It is well known that the planum temporale (PT) area in the posterior temporal lobe carries out spectro-temporal analysis of auditory stimuli, which is crucial for speech, for example. There are suggestions that the PT is also involved in auditory attention, specifically in the discrimination and selection of stimuli from the left and right ear. However, direct evidence is missing so far. To examine the role of the PT in auditory attention we asked fourteen participants to complete the Bergen Dichotic Listening Test. In this test two different consonant-vowel syllables (e.g., “ba” and “da”) are presented simultaneously, one to each ear, and participants are asked to verbally report the syllable they heard best or most clearly. Thus attentional selection of a syllable is stimulus-driven. Each participant completed the test three times: after their left and right PT (located with anatomical brain scans) had been stimulated with repetitive transcranial magnetic stimulation (rTMS), which transiently interferes with normal brain functioning in the stimulated sites, and after sham stimulation, where participants were led to believe they had been stimulated but no rTMS was applied (control). After sham stimulation the typical right ear advantage emerged, that is, participants reported relatively more right than left ear syllables, reflecting a left-hemispheric dominance for language. rTMS over the right but not left PT significantly reduced the right ear advantage. This was the result of participants reporting more left and fewer right ear syllables after right PT stimulation, suggesting there was a leftward shift in stimulus selection. Taken together, our findings point to a new function of the PT in addition to auditory perception: particularly the right PT is involved in stimulus selection and (stimulus-driven), auditory attention.

## Introduction

The planum temporale (PT) is located in the superior temporal gyrus just posterior to the primary auditory cortex (Heschl's gyrus). There is large consensus that the PT serves as a “computational hub” for complex sounds [1] and is a part of a language network that segregates and matches fine-grained spectro-temporal representations [2], [3], allowing the discrimination of auditory stimuli such as phonemes [4], [5], [6]. There are findings suggesting that beyond auditory perception the PT is additionally involved in or modulated by attentional processes [7], [8]. However, these findings are inconsistent and based on correlational approaches. The present study provided a direct test of the role of the PT by applying repetitive transcranial magnetic stimulation (rTMS) to this area, a technique that induces weak electrical currents from outside the skull via rapidly changing magnetic fields and temporarily interferes with the neuronal functioning of the stimulated area (for review [9]).

Dichotic listening, which today is mostly used for assessing language lateralization, was actually originally developed to study auditory attention [10], [11]. In the most commonly used version of the dichotic listening paradigm two different consonant vowel-syllables (e.g., da–ba) are presented simultaneously, that is, one to each ear [12]. When asked which syllable participants heard best or most clearly, they typically report the right ear stimulus [13], the so-called right ear advantage, which is commonly interpreted to indicate left hemispheric specialization for language processing (see [14]; for review [15]). There is ample, converging evidence that dichotic listening taps into PT functioning. For instance, complete callosotomy or lesions of the posterior portion of the corpus callosum almost completely abolishes reports of the stimuli presented to the left ear [16], [17], [18]. In a Diffusion Tensor Imaging fiber tracking study Westerhausen, Grüner, Specht and Hugdahl [19] accordingly found a positive correlation between the number of left ear reports and the thickness in the commissural tract that connects the PT areas of both hemispheres. Moreover, activations in the PT during dichotic listening is a very robust finding that has been replicated in many neuroimaging studies (e.g., [7], [20], [21], [22]). Furthermore, Pollmann et al. [16], [23], [24]) suggested that the PT plays a crucial role in stimulus selection during dichotic listening. Which of the two stimuli is reported depends on bottom-up and top-down attentional factors. For example, salience differences in the consonants' voice onset time ( = bottom-up processing) as well as which ear is (more) attended to ( = top-down processing) affect stimulus selection [25], [26]. Indeed, some dichotic listening studies found activations in the PT related to attentional processes [7], [27], [28], [29]. Similar attention-related activations were reported by other studies that did not use dichotic listening suggesting the activations are not dichotic listening specific (e.g., [30], [31]). As pointed out by Griffiths and Warren [1] and Hashimoto et al. [27], however, there are also reports that failed to show an involvement of the PT in attentional processes [32], [33], [34], [35]. All these studies used either functional magnetic resonance imaging [27], [28], [30], [31], [32], magnetoencephalography [29], or positron emission tomography [7], [33], [34], [35]. One might speculate that because the main function of the PT is spectro-temporal analysis, additional processes like stimulus selection are difficult to detect with neuroimaging, because both result in PT activations.

Clinical studies also failed to provide a clear answer as to whether the PT is involved in attention or stimulus selection. Karnath, Ferber and Himmelbach [36] argued that neglect arises from lesions of the superior temporal gyrus, but these findings have been disputed and the majority of researchers seem to agree on the inferior parietal lobule and temporo-parietal junction as epicenters of multimodal attentional processing (for a recent review [37]). In dichotic listening, the effects of unilateral lesions or epileptic foci seem to be relatively mild, although they become statistically significant on group level. For example, patients with unilateral left temporal lobectomy including Heschl's gyrus reported fewer left and right ear stimuli than controls, but the deficit was rather subtle implying that even with a lesioned primary auditory cortex stimuli from both ears can be processed [17]. The study did not, however, specify to what extent areas beyond Heschl's gyrus were affected, which illustrates a general problem with clinical findings – apart from inconsistent findings (e.g., [38], [39], [40]): lesions or epileptic foci are seldom confined to the PT making it difficult to determine the specific function of this area.

Taken together, a number of empirical findings suggest that beyond auditory perception the PT may play an active part in auditory attention. Particularly in dichotic listening, the PT is said to be crucial for stimulus selection [23]. Clinical and neuroimaging studies do not provide unequivocal evidence for or against this hypothesis. We therefore used anatomically guided rTMS in the present study to further elucidate the exact role of the PT in auditory attention in general, and in particular, in the dichotic listening situation. rTMS is relatively focal with a spatial resolution down to a few millimeters [41], and therefore allows direct examination of the specific role of the PT. Moreover, in contrast to neuroimaging which can only reveal which brain areas are *correlated* with dichotic listening, rTMS enabled us to investigate whether the left and right PT are, indeed, *required* for the right ear advantage. There are two types of dichotic listening paradigms: in the ‘forced attention’ paradigm participants are specifically instructed to report the left or right ear stimulus (top-down processing), while in the ‘nonforced’ paradigm participants report the stimulus they heard best or most clearly. Since ‘forced attention’ paradigms often lead to activations in the prefrontal and anterior cingulate cortex rather than in the PT [42], [43], we opted for a stimulus-driven, ‘nonforced’ paradigm here. In general, phonological processing in the PT is largely bilateral, though activations in the left PT are more pronounced during speech perception (e.g., [22]; for review [1]). There are hints that the attentional processes in the PT might be lateralized to the right hemisphere [23]. Our rationale was thus as follows: if stimulation of the PT (particularly of the left PT) interferes with phonological processing, then the general ability to report stimuli should be impaired (i.e., the *number* of reported syllables is generally reduced). If, however, the PT (particularly the right PT) is critical for (stimulus-driven) auditory attention, then rTMS over this region should lead to a shift in the *proportion* of left relative to right ear reports, while the total number of reported syllables should remain stable.

## Methods

### Ethical statement

Participants gave written informed consent in accordance with the Declaration of Helsinki. Approval of study was granted by the Regional Committees for Medical and Health Research Ethics (REK vest). Participants were recruited via advertisement and financially compensated for their participation.

### Participants

We tested 18 right-handed participants (9 women, 9 men). The mean age for women was 23.9 yrs (*SD*  = 2.9 yrs, range 20–29 yrs) and for men 24.0 yrs (SD = 4.0 yrs, range: 19–31 yrs). The participants' handedness was verified with the Edinburgh Handedness Inventory [44], which provides the degree of hand preference in percentage in a laterality quotient (LQ). Values range from –100 to +100 with negative values indicating a left- and positive values a right-hand preference. Women had a mean LQ of 96.5 (*SD* = 5.3, range 88.9–100), men a mean LQ of 91.4 (*SD* = 9.3, range 77.8–100, *t*(16) = 1.43, p = .172). Thus, both groups showed a strong right hand preference. Moreover, hearing thresholds in all participants were determined for frequencies of 250, 500, 1000, 2000, and 3000 Hz in both ears. In none of the participants mean hearing thresholds deviated more than 16dB from normed values and none had an interaural acuity difference greater than 12 dB. Participants were checked for TMS exclusion criteria [45], [46].

### Dichotic Listening

The Bergen Dichotic Listening Test comprises six consonant-vowel syllables (ba, da, ga, pa, ta, ka), which are presented dichotically via supra-aural headphones [13], [26]. The syllable of each stimulus pair (36 in total) are temporally aligned to ensure simultaneous onset of the left- and right-ear stimulus. Stimulus duration ranges between 350 and 450 ms depending on voice onset time differences between the different consonants. The 36 pairs were presented in a pseudorandomized order with an inter-stimulus interval of 4000 ms and participants were instructed to orally report the syllable they heard best or most clearly. The response was recorded by the experimenter with a scoring sheet. Three dependent variables were determined: (1) the accuracy rate (i.e., the percentage of correctly identified syllables) in the left (N_L_) and right ear (N_R_) disregarding the six homonymic pairs (e.g., ba–ba) which served as control stimuli; (2) the overall accuracy as a measure for the general phonological processing ability, calculated as: N_R_ + N_L_; (3) the LQ as a measure for ear asymmetry, calculated as: (N_R_–N_L_)/(N_R_+N_L_)*100. This formula controls for overall performance (cf. [47]) and, like in the Edinburgh Handedness Inventory above, produces values between +100 and -100, whereby positive values indicate a right ear/left-hemispheric advantage for language and negative values a left ear/right-hemispheric advantage.

### Magnetic Resonance Imaging

Prior to rTMS each participant underwent magnetic resonance imaging (MRI) to locate the left and right PT. MRI was performed on a 3T GE Signa platform and for all participants the imaging protocol consisted of a scout sequence followed by a structural, T1-weighted acquisition. The T1-weighted images were acquired with a Fast Spoiled Gradient sequence (repetition time, TR = 8.0 ms; echo time, TE = 3.2 ms, flip angle = 11 degrees) measuring 180 consecutive sagittal slices with field of view of 256 mm×256 mm and a 256×256 scan matrix, and a slice thickness of 1 mm. Thus, the image resolution (voxel size) was 1 mm×1 mm×1 mm.

### Magnetic stimulation

**Figure 1:**
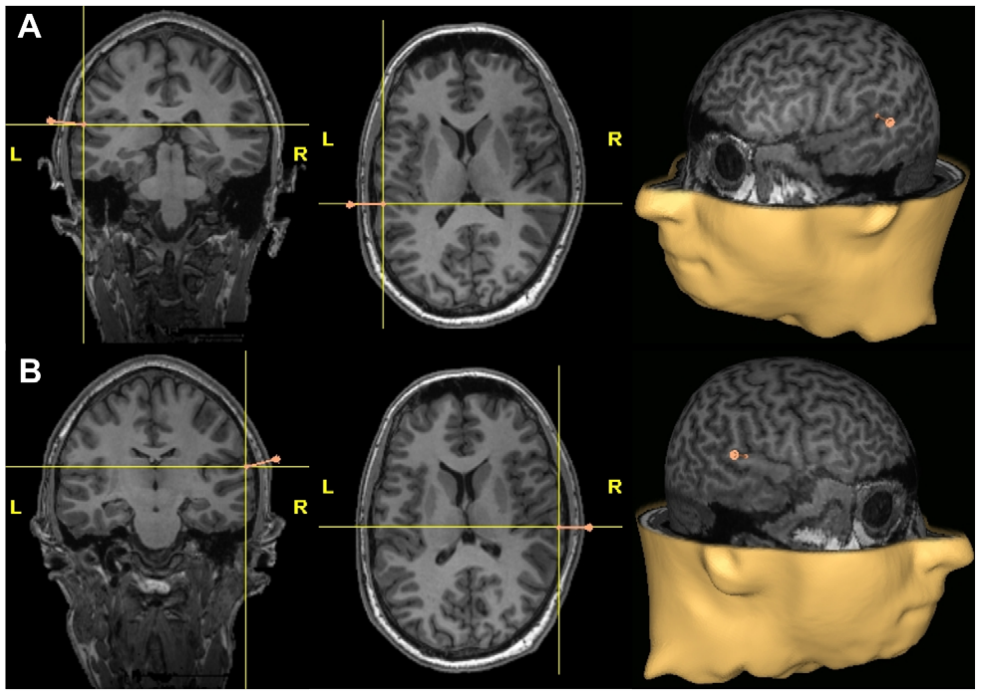
Stimulus sites. Location of the stimulation sites left (A) and right (B) planum temporale.

Each individual's T1-weighted brain scan and head were co-registered using frameless stereotaxy (eXimia NBS, NexStim Company, Helsinki, Finland). An example for the exact location of the stimulation sites can be seen in Figure 1.

To ensure that the clicking noise of the TMS coil does not interfere with the perception of the dichotic listening syllables an offline rTMS protocol was employed. Participants were told to close their eyes and relax while they were stimulated for 10 minutes at a frequency of 1 Hz ( = 600 pulses). This stimulation protocol affects the stimulated area for approximately 5 min *after* stimulation [9], [48]. As soon as the stimulation stopped, participants were given headphones and started the Bergen Dichotic Listening test.

Fourteen participants were stimulated with a MagPro X100 (Medtronic, Minneapolis, USA), while four participants were stimulated with a MagPro X100 (MagVenture, Farum, Denmark). The latter is an upgrade of the identical model and served as a temporary replacement due to maintenance works on the Medtronic stimulator. The electromagnetic output data indicated that the stimulation parameters of both stimulators were identical. Offline rTMS and sham stimulation were performed using an MCF-B65 figure-of-eight coil with 75 mm outer diameter and a MCF-P-B65 placebo coil, respectively (both MagVenture). The coils are identical in outer appearance and sound level, but the placebo coil has a built-in magnetic shield that reduces field strength by ca. 80%.

The stimulator output intensity was determined based on the individual motor threshold (MT) following the “best Parameter Estimation by Sequential Testing” protocol [49]. The MT is defined as the stimulator intensity that leads to motor evoked potentials (>50 µV) in half of the stimulations [45], [50]. The muscle activity of the abductor pollicis brevis was monitored with an EMG device (ME6000, Mega Electronics, Kuopio, Finland) while the corresponding motor cortex was stimulated. The stimulation intensity for the left and right PT was set at 110% of the MT of the contralateral hand with a predefined minimum of 45% and a maximum of 60% stimulator output. For instance, if the left hand had a MT = 46% and the right hand MT = 50% stimulator output, the right PT was stimulated with 51% (110% of 46) and the left PT with 55% (110% of 50) stimulator output. In four participants the hand area could not be located. Therefore, intensity was set either to 60% stimulator output or to the stimulator output of the contralateral hand. In two participants stimulator output was reduced from 60% to 55% to diminish facial twitches. The intensity during sham stimulation was identical to the stimulus output of real rTMS at the same site to have an identical sound level of the placebo coil (i.e., if the left PT was stimulated with 55% stimulator output, sham stimulation was also performed with 55%). Mean intensity for the left and right PT was 55.2% (*SD* = 4.2) and 57.4% (*SD* = 3.3, *t*(17)  = 2.42, *p* = .027) stimulus output, respectively.

### Procedure

After participants' hearing thresholds were determined, they underwent MRI to obtain anatomical scans. A few days later the rTMS session took place. After determining the MT, participants completed the Bergen Dichotic Listening test three times ( = three blocks): after stimulation of the left and right PT, and after sham stimulation (control). Between two blocks was a 30 minute “wash-out” period to ensure that neurons went back to baseline. Block order was counterbalanced across participants. In half of the participants the placebo coil was positioned on the left, in the other half on the right PT. We decided against two sham blocks for each participant, because this would have made the rTMS session longer than 3.5 h and we wanted to avoid fatigue effects.

## Results

Throughout effect sizes for ANOVAs and post-hoc t-tests are provided as the proportion of variance accounted for (partial η^2^) or as Cohen's d, respectively. To have sufficient test power for detecting significant effects with the typically small TMS sample, post hoc t-tests were conducted without α-error adjustment and an α -level of *p* = .05.

First we examined whether there was a difference between positioning the coil over the left and right PT during sham. Left and right ear accuracy rates from the sham condition were subjected to a 2×2×2 mixed ANOVA with Sex and Sham site (left, right) as between- and Ear (left, right) as within-participants effects. Only a main effect Ear emerged (*F*(1,14) = 5.84, *p*<.001, η^2^ = .29) with participants reporting more syllables from the right ear (all other *F*≤1.86, all other *p*≥.194). In subsequent analyses Sham site was therefore discarded. Moreover, examination of the sham condition revealed that 4 of the 18 participants (i.e., 17%) showed a left ear advantage. This is in alignment with a large dichotic listening dataset of N = 1800 (including both left- and right-handers), in which the proportion of participants with a left ear advantage is 22% [51]. These four participants are more likely to show right-hemispheric language lateralization [52] and differential lateralization effects of the left and right PT might be masked when left- and right-lateralized participants are pooled. Since a sample of n = 4 is also too small to be treated as a separate group, these participants were removed from subsequent analyses leaving 14 participants (7 male, 7 female).

To study whether stimulation of the PT affected the general ability to report stimuli, the overall performance was subjected to a 3×2 mixed ANOVA with Sex as between- and rTMS site (left, right, sham) as within-participants factor. No significant effects emerged (all *F*≤1.0, all *p*≥.516) and the overall performance in the three conditions was virtually identical (left PT: *M* = 90.2±*SE* = 1.7%, right PT: 91.4±2.0%, sham: 91.9±2.2%), suggesting that rTMS over the PT did not interfere with the general phonological processing ability.

**Figure 2:**
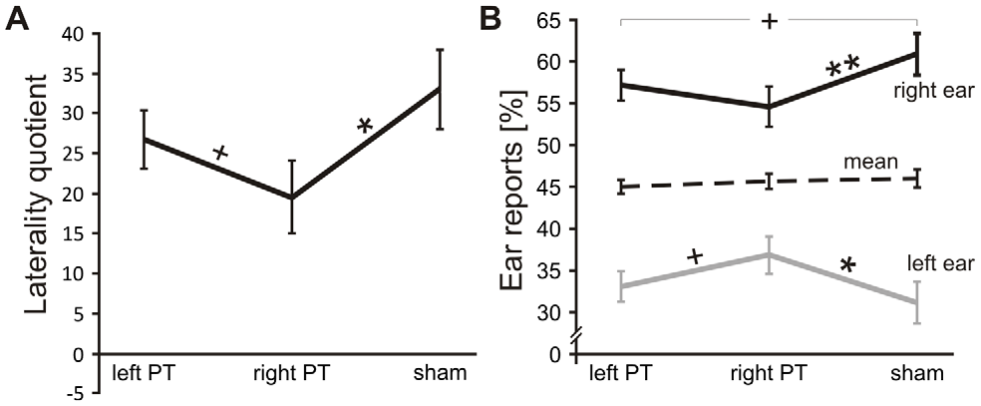
Effects of transcranial magnetic stimulation on planum temporale during dichotic listening. (A) Laterality quotient (±*SE*) across left PT, right PT, and sham stimulation. Positive values indicate a left, negative a right ear advantage. Note the reduced right ear advantage after right PT stimulation. (B) Percentage of reported syllables (±*SE*) from the left and right ear across left PT, right PT and sham stimulation. The dashed line represents the mean of left and right ear reports. Note that right PT stimulation alters the proportion of left and right ear reports while the mean remains stable. **p<.01, *p<.05, **^+^** *p*<.10

To examine whether PT stimulation affected ear asymmetry (i.e., the proportion of left and right ear reports) the 3×2 mixed ANOVA was repeated with the LQ as dependent variable. Unsurprisingly, the intercept became significant (*F*(1,12) = 53.43, *p*<.001, η^2^ = .82) with an overall positive LQ (26.3±3.6) indicating a right ear advantage. Moreover, a main effect of rTMS site emerged (*F*(2,24) = 4.71, *p* = .019, η^2^ = .28). Post hoc comparisons showed that after rTMS over the right PT the right ear advantage was significantly reduced compared with sham (*t*(13) = 2.83, *p* = .014, *d* = 0.76) and left PT stimulation (*t*(13) = 1.87, *p* = .08, *d* = 0.50), although the latter only showed a trend (see Figure 2A). No significant difference was found between left PT stimulation and sham (*t*(13) = 1.34, *p* = .192, *d* = 0.37) and no further main effect or interaction reached significance (all *F*≤1.0, all *p*≥.423).

The reduced right ear advantage after right PT stimulation may have been the result of reduced left ear reports, increased right ear reports or both, that is, a shift from right to left ear reports. To further investigate this, we carried out an additional 3×2×2 mixed ANOVA with Sex as between- and rTMS site (left, right, sham) as well as Ear (left, right) as within-participants factors with left and right ear accuracy as dependent variables. The main effect Ear (*F*(1,24) = 53.36, *p*<.001, η^2^ = .82) merely reflected the intercept in the previous ANOVA with more reports from the right (*M* = 57.5±*SE* = 1.9%) than left ear (33.6±1.8%). The main effect of TMS did not become significant (*F*(2,24)<1.0, *p* = .589) reflecting that the overall performance was similar in the three conditions (left PT: 45.1±0.8%, right PT: 45.7±1.0%, sham: 46.0±1.1%). In accordance with the LQ ANOVA, the interaction between Ear and rTMS site was significant (*F*(2,24) = 4.70, *p* = .019, η^2^ = .28). The right ear advantage was substantially lower after right PT stimulation (left PT: Cohen's *d* = 2.07, right PT: *d* = 1.17, sham: *d* = 1.76), although post hoc t-tests revealed a significant right ear advantage in all three conditions (all *t*(13)≥4.38, all *p*≤.001). Crucially, when comparing right PT stimulation with sham, significantly fewer syllables were reported from the right ear (right PT: 54.5±2.3%, sham: 61.0±2.6%, *t*(13) = 3.09, *p* = .009, *d* = 0.83) *and* significantly more syllables were reported from the left ear (right PT: 36.9±2.2%, sham: 31.0±2.5%, *t*(13) = 2.53, *p* = .025, *d* = 0.68). This indicates a shift from right to left ear responses. The other comparisons failed to reach significance, although there was a trend for more *right* ear reports after sham (61.0±2.6%) as compared with left PT stimulation (57.1±1.7%, *t*(13) = 2.00, *p* = .067, *d* = 0.53) and a trend for more *left* ear reports after right PT stimulation (36.9±2.2%) as compared with left PT stimulation (33.1±1.8%, *t*(13) = 1.9, *p* = .080, *d* = 0.51). For an overview of the interaction and significant effects see Figure 2B. No other main effect or interaction reached significance (all *F*≤1.0, all *p*≥.516).

## Discussion

The aim of the present study was to examine whether the PT is involved in auditory attention and stimulus selection (e.g., [23], [27]). First of all, we found the characteristic right ear advantage and a typical proportion of participants with a left ear advantage implying that the role of the PT was examined in a typical sample. Crucially, the results revealed that offline, low-frequency rTMS over the right PT significantly reduced the magnitude of the right ear advantage as compared with sham. A more detailed analysis showed that participants reported fewer syllables from the right and more syllables from the left ear. Together with the finding that the general ability to report stimuli remained stable, this finding suggests that the right PT stimulation induced an attentional shift towards left ear stimuli. This is consistent with Pollmann's [23] idea that the right PT is critical for stimulus selection in dichotic listening and demonstrates that beyond spectro-temporal analyses and phonological processing the PT is also involved in (stimulus-driven), auditory attention (see also [7], [28], [30]). It is also consistent with the idea that the PT is part of postero-dorsal phonological processing stream that analyzes attentional and spatial components of speech [2]. Another rTMS study [53] stimulated the right posterior superior temporal gyrus, which largely overlaps with the PT area, and the right posterior parietal cortex during a visual exploratory search task. Ellison et al. [53] found a double dissociation: Online rTMS over the right posterior superior temporal gyrus affected visual search that required finding items based on orientation and color. However, rTMS over the right posterior parietal cortex impaired visual search based on shapes. The findings are important in two ways. First, the (right) PT area does not only seem to respond to auditory but also visual stimuli, suggesting attentional processing in this area might, in fact, be multimodal. Second, the fact that a double dissociation arose, implies that rTMS over the PT area did not spread to parietal areas, which are known to be critical for attention and damage to these areas leads to neglect [54].Thus, although we cannot entirely rule out the possibility, it seems rather unlikely that the effect we found for the right PT, is in fact the consequence of activation spreading to the inferior parietal/temporo-parietal junction regions.

We suggest that the PT is part of a right lateralized attentional network comprising the temporo-parietal and inferior frontal cortex [55], and depending on specific task demands the PT's attentional processing capacities get involved or not. According to Corbetta and Shulman [55] this network chiefly deals with bottom-up rather than top-down processing, which is why we opted for a ‘nonforced’ dichotic listening paradigm to probe the attentional capacities of the PT (i.e., instructing participants to report the sound they heard best or most clearly). As pointed out above, top-down processing is often associated with activations in the prefrontal and anterior cingulate cortex [42], [43], presumably because the ‘forced’ paradigm largely relies on executive control functions [56], and thus seemed less suitable to probe the role of the PT in auditory attention. Nevertheless, it would be interesting to examine in the future whether the PT also plays a role in top-down processing, that is, in a ‘forced attention’ paradigm.

At first, the facilitatory effect of rTMS might be surprising, since offline, low-frequency rTMS is often described as having inhibitory, functional effects. One might thus have expected that the right ear advantage would have been *increased* after rTMS over the right PT, rather than decreased as shown in the present study. However, inhibitory rTMS effects are well documented for stimulating primary motor areas and these findings cannot simply be generalized to other brain regions. In fact, several authors have demonstrated that offline, low-frequency rTMS can very well have facilitatory effects [57], [58], [59]. Accordingly, patients with epileptic foci also demonstrated facilitatory effects in dichotic listening ([39], [40]; but see [38]). Our results are thus not at odds with the literature and our rTMS protocol seemed to have facilitated right PT functioning.

Although the results were straightforward with respect to the right PT, findings on the role of the left PT were less clear. We hypothesized that stimulation of the left PT in particular would impair the general ability to report consonant-vowel syllables. However, while numerically the overall accuracy rate was lowest after left PT stimulation there were no significant differences between any of the three stimulation conditions. In general, stimulation of the left PT did not lead to significant effects, only trends emerged. From Figure 2 it appears as if stimulation of the left PT reduced the right ear advantage a little: not enough to result in a significant difference with sham, but enough so that the difference between left and right PT stimulation only showed a trend.

The finding that left (and right) PT stimulation did not affect the general ability to report syllables does not argue against the notion that the PT carries out spectro-temporal analysis and phonological processing. Other, more parsimonious explanations may account for the lack of effect. First, as noted above unilateral temporal lesions have relatively little effect on reporting left and right ear stimuli in dichotic listening [17]. This might reflect that auditory processing, in contrast to visual processing, for example, is largely bilaterally organized, so that unilateral lesions or rTMS have little effect on a basic processing level. Alternatively, rTMS might be too focal to exert meaningful effects on the left PT. The activation in the PT during dichotic listening is typically stronger and more widespread in the left than right hemisphere [13]. Moreover, the PT shows a marked anatomical asymmetry with the left PT being larger than the right (e.g., [60], [61]; for review [62]). It is thus possible that the left PT area was simply too large for the relatively focal rTMS. Finally, one might, perhaps, speculate that the differential effects of left and right PT stimulation arise from the fact that stimulation over the right PT was slightly stronger on average than over the left PT (57% versus 55% stimulator output). However, although the difference of 2% was statistically significant, it is so small that it is very unlikely to have any practical consequences. We thus do not believe that it can account for the finding that left PT stimulation did not show any meaningful behavioral effects.

The relative lack of left PT stimulation, however, implies that there is no interhemispheric spread of activation effect. rTMS cannot only lead to concurrent, electrical activations in areas adjacent to stimulated brain sites but also in homotopic areas in the contralateral hemisphere [63]. This might explain the somewhat reduced right ear advantage after left PT stimulation, but it does not explain that only right PT stimulation resulted in a reduced right ear advantage compared with sham. Hence, it can be argued that the attentional shift is a genuine effect of right PT stimulation and not the result of interhemispheric spread of activation.

Taken together, the findings of the present study show that the right PT plays a crucial role in stimulus selection during dichotic listening [23]. This lends very strong support to the notion that at least the right PT is involved in (stimulus-driven), auditory attention. We did not find any indication that rTMS over the PT impairs the general ability to report consonant-vowel syllables. This might be the result of the bilateral organization of the auditory system or, perhaps, that rTMS over the left PT is too focal to exert meaningful effects. Nevertheless, the PT appears to be involved in bottom-up attentional (seemingly multimodal) processing, probably as part of a network comprising temporo-parietal and inferior frontal areas [55].

## References

[1] Griffiths TD, Warren JD (2002) The planum temporale as a computational hub. Trends in Neurosciences 25: 348–353. 12079762 10.1016/s0166-2236(02)02191-4

[2] Rauschecker JP, Scott SK (2009) Maps and streams in the auditory cortex: nonhuman primates illuminate human speech processing. Nature Neuroscience 12: 718–724. 19471271 10.1038/nn.2331 PMC2846110

[3] Hickok G, Poeppel D (2007) Opinion - The cortical organization of speech processing. Nature Reviews Neuroscience 8: 393–402. 17431404 10.1038/nrn2113

[4] Rimol LM, Specht K, Weis S, Savoy R, Hugdahl K (2005) Processing of sub-syllabic speech units in the posterior temporal lobe: An fMRI study. Neuroimage 26: 1059–1067. 15894493 10.1016/j.neuroimage.2005.03.028

[5] Specht K, Reul J (2003) Functional segregation of the temporal lobes into highly differentiated subsystems for auditory perception: an auditory rapid event-related fMRI-task. Neuroimage 20: 1944–1954. 14683700 10.1016/j.neuroimage.2003.07.034

[6] Wise RJS, Scott SK, Blank SC, Mummery CJ, Murphy K, et al (2001) Separate neural subsystems within 'Wernicke's area'. Brain 124: 83–95. 11133789 10.1093/brain/124.1.83

[7] O'Leary DS, Andreasen NC, Hurtig RR, Hichwa RD, Watkins GL, et al (1996) A positron emission tomography study of binaurally and dichotically presented stimuli: Effects of level of language and directed attention. Brain and Language 53: 20–39. 8722897 10.1006/brln.1996.0034

[8] Woldorff MG, Gallen CC, Hampson SA, Hillyard SA, Pantev C, et al (1993) Modulation of early sensory processing in human auditory cortex during auditory selective attention. Proceedings of the National Academy of Sciences USA 90: 8722–8726. 10.1073/pnas.90.18.8722 PMC47430 8378354

[9] Walsh V, Pascual-Leone A (2003) Transcranial magnetic stimulation: a neurochronometrics of mind. Cambridge: The MIT Press.

[10] Broadbent DE (1954) The role of auditory localization in attention and memory span. Journal of Experimental Psychology 47: 191–196. 13152294 10.1037/h0054182

[11] Broadbent DE (1952) Listening to one of two synchronous messages. Journal of Experimental Psychology 44: 51–55. 14955590 10.1037/h0056491

[12] Studdert-Kennedy M, Shankweiler D (1970) Hemispheric specialization for speech perception. Journal of the Acoustical Society of America 48: 579–594. 5470503 10.1121/1.1912174

[13] Hugdahl K (1995) Dichotic listening: probing temporal lobe functional integrity. In: Davidson RJ, Hugdahl K, editors. Brain Asymmetry. Cambridge, MA: MIT Press. pp. 123–156.

[14] Kimura D (1967) Functional asymmetry of the brain in dichotic listening. Cortex 3: 163–178.

[15] Westerhausen R, Hugdahl K (2008) The corpus callosum in dichotic listening studies of hemispheric asymmetry: A review of clinical and experimental evidence. Neuroscience and Biobehavioral Reviews 32: 1044–1054. 18499255 10.1016/j.neubiorev.2008.04.005

[16] Pollmann S, Maertens M, von Cramon DY, Lepsien J, Hugdahl K (2002) Dichotic listening in patients with splenial and nonsplenial callosal lesions. Neuropsychology 16: 56–64. 11858226 10.1037//0894-4105.16.1.56

[17] Milner B, Taylor L, Sperry RW (1968) Lateralized suppression of dichotically presented digits after commissural section in man. Science 161: 184–186. 5657069 10.1126/science.161.3837.184

[18] Sparks R, Geschwind N (1968) Dichotic listening in man after section of neocortical commissures. Cortex 4: 3–16.

[19] Westerhausen R, Gruner R, Specht K, Hugdahl K (2009) Functional Relevance of Interindividual Differences in Temporal Lobe Callosal Pathways: A DTI Tractography Study. Cerebral Cortex 19: 1322–1329. 18842665 10.1093/cercor/bhn173

[20] van den Noort M, Specht K, Rimol LM, Ersland L, Hugdahl K (2008) A new verbal reports fMRI dichotic listening paradigm for studies of hemispheric asymmetry. Neuroimage 40: 902–911. 18234509 10.1016/j.neuroimage.2007.11.051

[21] Jäncke L, Specht K, Shah JN, Hugdahl K (2003) Focused attention in a simple dichotic listening task: an fMRI experiment. Cognitive Brain Research 16: 257–266. 12668235 10.1016/s0926-6410(02)00281-1

[22] Hugdahl K, Bronnick K, Kyllingsbaek S, Law I, Gade A, et al (1999) Brain activation during dichotic presentations of consonant-vowel and musical instrument stimuli: a O-15-PET study. Neuropsychologia 37: 431–440. 10215090 10.1016/s0028-3932(98)00101-8

[23] Pollmann S (2010) A unified structural-attentional framework for dichotic listening. In: Hugdahl K, Westerhausen R, editors. The two halves of the brain – information processing in the cerebral hemispheres Cambridge, MA: MIT Press. pp. 441–468.

[24] Pollmann S, Maertens M, von Cramon DY (2004) Splenial lesions lead to supramodal target detection deficits. Neuropsychology 18: 710–718. 15506839 10.1037/0894-4105.18.4.710

[25] Rimol LM, Eichele T, Hugdahl K (2006) The effect of voice-onset-time on dichotic listening with consonant–vowel syllables. Neuropsychologia 44: 191–196. 16023155 10.1016/j.neuropsychologia.2005.05.006

[26] Hugdahl K, Andersson L (1986) The forced-attention paradigm in dichotic-listening to cv-syllables - a comparison between adults and children. Cortex 22: 417–432. 3769494 10.1016/s0010-9452(86)80005-3

[27] Hashimoto R, Homae F, Nakajima K, Miyashita Y, Sakai KL (2000) Functional differentiation in the human auditory and language areas revealed by a dichotic listening task. Neuroimage 12: 147–158. 10913321 10.1006/nimg.2000.0603

[28] Pugh KR, Shaywitz BA, Fulbright RK, Byrd D, Skudlarski P, et al (1996) Auditory selective attention: An fMRI investigation. Neuroimage 4: 159–173. 9345506 10.1006/nimg.1996.0067

[29] Alho K, Salonen J, Rinne T, Medvedev SV, Hugdahl K, et al (2012) Attention-related modulation of auditory-cortex responses to speech sounds during dichotic listening. Brain Research 1442: 47–54. 22300726 10.1016/j.brainres.2012.01.007

[30] Downar J, Crawley AP, Mikulis DJ, Davis KD (2000) A multimodal cortical network for the detection of changes in the sensory environment. Nature Neuroscience 3: 277–283. 10700261 10.1038/72991

[31] Hall DA, Haggard MP, Akeroyd MA, Summerfield AQ, Palmer AR, et al (2000) Modulation and task effects in auditory processing measured using fMRI. Hum Brain Mapp 10: 107–119. 10912590 10.1002/1097-0193(200007)10:3<107::AID-HBM20>3.0.CO;2-8 PMC6871907

[32] Maeder PP, Meuli RA, Adriani M, Bellmann A, Fornari E, et al (2001) Distinct pathways involved in sound recognition and localization: A human fMRI study. Neuroimage 14: 802–816. 11554799 10.1006/nimg.2001.0888

[33] Zatorre RJ, Mondor TA, Evans AC (1999) Auditory attention to space and frequency activates similar cerebral systems. Neuroimage 10: 544–554. 10547331 10.1006/nimg.1999.0491

[34] Tzourio N, ElMassioui F, Crivello F, Joliot M, Renault B, et al (1997) Functional anatomy of human auditory attention studied with PET. Neuroimage 5: 63–77. 9038285 10.1006/nimg.1996.0252

[35] Frith CD, Friston KJ (1996) The role of the thalamus in ''top down'' modulation of attention to sound. Neuroimage 4: 210–215. 9345511 10.1006/nimg.1996.0072

[36] Karnath HO, Ferber S, Himmelbach M (2001) Spatial awareness is a function of the temporal not the posterior parietal lobe. Nature 411: 950–953. 11418859 10.1038/35082075

[37] Jacobs S, Brozzoli C, Farne A (2012) Neglect: A multisensory deficit? Neuropsychologia 50: 1029–1044. 22465475 10.1016/j.neuropsychologia.2012.03.018

[38] Lee GP, Loring DW, Varney NR, Roberts RJ, Newell JR, et al (1994) Do dichotic word listening asymmetries predict side of temporal-lobe seizure onset. Epilepsy Research 19: 153–160. 7843170 10.1016/0920-1211(94)90025-6

[39] Mazzucchi A, Visintini D, Magnani G, Cattelani R, Parma M (1985) Hemispheric prevalence changes in partial epileptic patients on perceptual and attentional tasks. Epilepsia 26: 379–390. 3930231 10.1111/j.1528-1157.1985.tb05668.x

[40] Mazzucchi A, Parma M (1978) Responses to dichotic-listening tasks in temporal epileptics with or without clinically evident lesions. Cortex 14: 381–390. 710148 10.1016/s0010-9452(78)80064-1

[41] Cowey A (2005) The Ferrier Lecture 2004 what can transcranial magnetic stimulation tell us about how the brain works? Philosophical transactions of the Royal Society of London Series B, Biological sciences 360: 1185–1205. 16147516 10.1098/rstb.2005.1658 PMC1569499

[42] Thomsen T, Rimol LM, Ersland L, Hugdahl K (2004) Dichotic listening reveals functional specificity in prefrontal cortex: an MRI study. Neuroimage 21: 211–218. 14741658 10.1016/j.neuroimage.2003.08.039

[43] Jäncke L, Buchanan TW, Lutz K, Shah NJ (2001) Focused and nonfocused attention in verbal and emotional dichotic listening: An FMRI study. Brain and Language 78: 349–363. 11703062 10.1006/brln.2000.2476

[44] Oldfield RC (1971) Assessment and Analysis of Handedness - Edinburgh Inventory. Neuropsychologia 9: 97–113. 5146491 10.1016/0028-3932(71)90067-4

[45] Rossi S, Hallett M, Rossini PM, Pascual-Leone A (2009) Safety of TMS Consensus Group (2009) Safety, ethical considerations, and application guidelines for the use of transcranial magnetic stimulation in clinical practice and research. Clinical Neurophysiology 120: 2008–2039. 19833552 10.1016/j.clinph.2009.08.016 PMC3260536

[46] Wassermann EM (1998) Risk and safety of repetitive transcranial magnetic stimulation: report and suggested guidelines from the international workshop on the safety of repetitive transcranial magnetic stimulation, June 5–7, 1996. Evoked Potentials-Electroencephalography and Clinical Neurophysiology 108: 1–16. 10.1016/s0168-5597(97)00096-8 9474057

[47] Birkett P (1977) Measures of Laterality and Theories of Hemispheric Processes. Neuropsychologia 15: 693–696. 896026 10.1016/0028-3932(77)90075-6

[48] Hirnstein M, Bayer U, Ellison A, Hausmann M (2011) TMS over the left angular gyrus impairs the ability to discriminate left from right. Neuropsychologia 49: 29–33. 21035475 10.1016/j.neuropsychologia.2010.10.028

[49] Awiszus F, Borckardt JJ TMS Motor Threshold Assessment Tool (MTAT 2.0). Available: http://www.clinicalresearcher.org/software.htm. Accessed 2013 Jan 25.

[50] Rossini PM, Barker AT, Berardelli A, Caramia MD, Caruso G, et al (1994) Non-invasive electrical and magnetic stimulation of the brain, spinal cord and roots: basic principles and procedures for routine clinical application. Report of an IFCN committee. Electroencephalography and Clinical Neurophysiology 91: 79–92. 7519144 10.1016/0013-4694(94)90029-9

[51] Hirnstein M, Westerhausen R, Korsnes MS, Hugdahl K (2013) Sex differences in language asymmetry are age-dependent and small: A large-scale, consonant-vowel dichotic listening study with behavioral and fMRI data. In press 10.1016/j.cortex.2012.08.002 22980918

[52] Hugdahl K, Carlsson G, Uvebrant P, Lundervold AJ (1997) Dichotic-listening performance and intracarotid injections of amobarbital in children and adolescents - Preoperative and postoperative comparisons. Archives of Neurology 54: 1494–1500. 9400358 10.1001/archneur.1997.00550240046011

[53] Ellison A, Schindler I, Pattison LL, Milner AD (2004) An exploration of the role of the superior temporal gyrus in visual search and spatial perception using TMS. Brain 127: 2307–2315. 15292055 10.1093/brain/awh244

[54] Vallar G, Perani D (1986) The anatomy of unilateral neglect after right-hemisphere stroke lesions - a clinical CT-scan correlation study in man. Neuropsychologia 24: 609–622. 3785649 10.1016/0028-3932(86)90001-1

[55] Corbetta M, Shulman GL (2002) Control of goal-directed and stimulus-driven attention in the brain. Nature Reviews Neuroscience 3: 201–215. 11994752 10.1038/nrn755

[56] Kompus K, Specht K, Ersland L, Juvodden HT, van Wageningen H, et al (2012) A forced-attention dichotic listening fMRI study on 113 subjects. Brain and Language 121: 240–247. 22494771 10.1016/j.bandl.2012.03.004

[57] Silvanto J, Muggleton N, Walsh V (2008) State-dependency in brain stimulation studies of perception and cognition. Trends in Cognitive Sciences 12: 447–454. 18951833 10.1016/j.tics.2008.09.004

[58] Siebner HR, Lang N, Rizzo V, Nitsche MA, Paulus W, et al (2004) Preconditioning of low-frequency repetitive transcranial magnetic stimulation with transcranial direct current stimulation: Evidence for homeostatic plasticity in the human motor cortex. Journal of Neuroscience 24: 3379–3385. 15056717 10.1523/JNEUROSCI.5316-03.2004 PMC6730024

[59] Beaton AA (1997) The relation of planum temporale asymmetry and morphology of the corpus callosum to handedness, gender, and dyslexia: A review of the evidence. Brain and Language 60: 255–322. 9344480 10.1006/brln.1997.1825

[60] Dos Santos Sequeira S, Woerner W, Walter C, Kreuder F, Lueken U, et al (2006) Handedness, dichotic-listening ear advantage, and gender effects on planum temporale asymmetry—A volumetric investigation using structural magnetic resonance imaging. Neuropsychologia 44: 622–636. 16098999 10.1016/j.neuropsychologia.2005.06.014

[61] Geschwind N, Levitsky W (1968) HUMAN BRAIN - LEFT-RIGHT ASYMMETRIES IN TEMPORAL SPEECH REGION. Science 161: 186–187. 5657070 10.1126/science.161.3837.186

[62] van den Bos R, Homberg J, de Visser L (2013) A critical review of sex differences in decision-making tasks: Focus on the Iowa Gambling Task. Behavioural Brain Research 238: 95–108. 23078950 10.1016/j.bbr.2012.10.002

[63] Komssi S, Kahkonen S (2006) The novelty value of the combined use of electroencephalography and transcranial magnetic stimulation for neuroscience research. Brain Research Reviews 52: 183–192. 16545462 10.1016/j.brainresrev.2006.01.008

